# High frequency of autoimmune thyroiditis in euthyroid girls with premature adrenarche

**DOI:** 10.3389/fped.2023.1064177

**Published:** 2023-03-16

**Authors:** Eleni Magdalini Kyritsi, Ioannis-Anargyros Vasilakis, Ioanna Kosteria, Aimilia Mantzou, Alexandros Gryparis, Eva Kassi, Gregory Kaltsas, Christina Kanaka-Gantenbein

**Affiliations:** ^1^ Division of Endocrinology, Diabetes and Metabolism, First Department of Pediatrics, Medical School, National and Kapodistrian University of Athens, Aghia Sophia Children's Hospital, Athens, Greece; ^2^ Department of Endocrinology, Growth and Development, P. & A. Kyriakou Children's Hospital, Athens, Greece; ^3^ Department of Speech and Language Therapy, University of Ioannina, Ioannina, Greece; ^4^ Department of Biological Chemistry, Medical School, National and Kapodistrian University of Athens, Athens, Greece; ^5^ Endocrine Unit, 1st Department of Propaedeutic Internal Medicine, “Laiko” General Hospital, Medical School, National and Kapodistrian University of Athens, Athens, Greece

**Keywords:** autoimmune thyroiditis, premature adrenarche, hyperinsulinemia, androgens, leptin

## Abstract

**Objective:**

The purpose of this study was to investigate the frequency of autoimmune thyroiditis (AT) among euthyroid prepubertal girls presenting with premature adrenarche (PA). We also aimed to identify the clinical, metabolic, and endocrine profile of girls with AT and concurrent PA and compare them to girls with AT without PA, PA alone and healthy controls.

**Methods:**

Ninety-one prepubertal girls aged 5–10 years, who attended our department for AT, PA and normal variants of growth and puberty were recruited for the study: 73 girls had PA, 6 AT without PA and 12 were referred for investigation of growth. All girls underwent clinical examination, detailed biochemical and hormonal screen. Standard dose Synachten stimulation test (SDSST) and oral glucose tolerance test (OGTT) were performed in all girls with PA. The whole study population was divided in 4 groups: Group PA−/AT+ included 6 girls with AT without PA; Group PA+/AT− PA subjects without AT; Group PA+/AT+ girls with PA and concomitant AT; Group PA-/AT- twelve healthy girls without PA nor AT (controls).

**Results:**

Among 73 girls presenting with PA 19 had AT (26%). BMI, systolic blood pressure (SBP) and the presence of goiter significantly differed between the four groups (*p* = 0.016, *p* = 0.022 and *p* < 0.001, respectively). When comparing hormonal parameters among the four groups significant differences were found in leptin (*p* = 0.007), TSH (*p* = 0.044), anti-TPO (*p* = 0.002), anti-TG (*p* = 0.044), IGF-BP1 (*p* = 0.006), *Δ*4-*Α* (*p* = 0.01), DHEA-S (*p *= <0.001), IGF-1 (*p* = 0.012) and IGF-BP3 (*p* = 0.049) levels. TSH levels were significantly higher in Group PA+/AT+ compared to PA+/AT− and PA−/AT− (*p* = 0.043 and *p* = 0.016, respectively). Moreover, girls with AT (Groups PA−/AT+ and PA+/AT+) had higher TSH levels than those in Group PA+/AT- (*p* = 0.025). Girls in Group PA+/AT + showed higher cortisol response at 60 min post-SDSST than girls in Group PA+/AT− (*p* = 0.035). During the OGTT, insulin concentrations at 60 min were significantly higher in Group PA+/AT + compared to Group PA+/AT− (*p* = 0.042).

**Conclusion:**

A high frequency of AT among euthyroid prepubertal girls with PA was observed. The combination of PA with AT even in euthyroid state may be associated with a greater degree of insulin resistance, than PA alone.

## Introduction

Autoimmune thyroiditis (AT) is the most common organ-specific autoimmune disease, affecting 2%–5% of the general population with a strong female preponderance (i.e., women 5%–15% and men 1%–5%) (1). The pathogenesis of AT involves a complex interplay between environmental factors and genetic background, with up to 50% of cases having a first-degree relative with positive antithyroid antibodies (2–6). The prevalence of AT in children and adolescents ranges between 1.2%–9.6% (depending on the diagnostic criteria, age, sex, pubertal stage, ethnicity, and iodine status of the population studied), occurring rarely in children younger than 3 years and reaching a peak in early to mid-puberty. A female predominance of up to 6:1 has been observed (6, 7).

Accumulating evidence suggests an association between thyroid autoimmunity, obesity and cardiometabolic risk factors (8–10), whereas, elevated thyroid autoantibodies have been observed in an average of 22.3% of patients with PCOS compared with an average of 8.5% in healthy women (11). PCOS is a heterogeneous endocrine disorder affecting 5%–20% of women of reproductive age worldwide (12). It is characterized mainly by clinical and/or biochemical hyperandrogenism, ovulatory dysfunction and polycystic ovarian morphology and is associated with multiple metabolic aberrations, including insulin resistance and hyperinsulinemia, an increased risk of glucose intolerance and type 2 diabetes mellitus, dyslipidemia, hypertension, and endothelial dysfunction, independent of body mass index (BMI) (12, 13). It has been speculated that the combination of PCOS with AT, even in the euthyroid state, may be associated with more pronounced metabolic derangements than either of these conditions alone, although an underlying mechanism has not been defined (11, 14–16).

Results from previous studies underline the early developmental origin of PCOS, suggesting that premature adrenarche (PA) may represent a forerunner of this condition (17). PA refers to the appearance of clinical signs of androgen action before the age of 8 years in girls or 9 years in boys associated with adrenal androgen precursors concentrations high for the prepubertal chronological age, but appropriate for Tanner pubertal stage II–III, in the absence of central puberty, congenital adrenal hyperplasia due to steroidogenic enzyme defects, androgen producing tumors and exogenous source of androgens (18, 19). Traditionally, PA has been considered to represent a benign variant of pubertal development, however, several studies have revealed an association between PA and intrauterine growth retardation, being born small for gestational age (SGA), insulin resistance and components of the metabolic syndrome, as well as higher incidence of functional ovarian hyperandrogenism, that may precede the development of PCOS in these girls in adolescence or later in young adulthood (19–24).

The aim of the present study was to investigate the frequency of AT among euthyroid prepubertal girls presenting with PA in the outpatient setting. We also aimed to identify clinical, metabolic, and endocrine characteristics of euthyroid girls with AT and concurrent PA and compare them to those of prepubertal euthyroid girls with AT but no PA, PA alone and healthy controls.

## Materials and methods

### Subjects

Ninety-one prepubertal girls 5–10 years of age, who attended the Division of Endocrinology, Metabolism and Diabetes of the First Department of Pediatrics, National and Kapodistrian University of Athens Medical School at the Aghia Sophia Children's Hospital for AT, PA and normal variants of growth and puberty (**i.e., isolated premature thelarche**) between 2020 and 2022 were recruited for the study: 73 girls presented with or had a history of PA, 6 had AT without PA and 12 were referred to our Department for investigation of growth. **The latter showed a normal prepubertal growth rate, growing along the lower height percentiles within or slightly below their genetic potential, with a predicted final height falling within 2 standard deviations (∼8.5 cm) above and below their mid-parental height, based on the bone age. Baseline biochemical and hormonal investigations were within normal range in all cases, Taken together, these 12 girls were considered as having a variant of normal growth.** PA was defined as the appearance of any clinical sign(s) of adrenarche (pubic/axillary hair, oily hair/skin, adult-type body odor) before 8 years of age together with elevated concentrations of adrenal androgens in the absence of central puberty or other causes of androgen excess. Congenital adrenal hyperplasia was ruled out in all PA cases based on normal responses of cortisol and 17-hydroxyprogesterone (17-OHP) following a standard dose Synachten stimulation test (SDSST). AT diagnosis was made when at least two of the following criteria were met: i) **presence of subclinical hypothyroidism defined by modestly elevated thyroid stimulating hormone (TSH) levels (5–10 IU/ml) with normal concentrations of free thyroxine FT4 or overt hypothyroidism, as assessed by a an elevated TSH with a low FT4** (**6**)**;** ii) positive serum anti-thyroid peroxidase (anti-TPO) and/or anti-thyroglobulin (anti-TG) antibodies; and iii) typical sonographic features of AT (diffuse or irregular hypoechogenicity of the thyroid parenchyma) (25, 26). Out of 73 girls with PA: **2 had transient congenital hypothyroidism and had stopped levothyroxine (LT4) replacement therapy more than one year before study entry, showed negative thyroid autoantibodies and normal ultrasonographic findings of the thyroid (thus, no evidence of AT). Another 6 had subclinical hypothyroidism without the concomitant features of autoimmune thyroid disease and were euthyroid under levothyroxine treatment.** Two out of 6 girls with AT **but no PA** were also receiving levothyroxine substitution therapy. Girls who had abnormal TSH and/or FT4 levels were excluded from the study.

Birth weight, gestational age, pregnancy, perinatal, personal, and detailed family history for thyroid disorders, diabetes, PCOS, obesity, dyslipidemia, and cardiovascular disease was obtained from all participants.

### Clinical evaluation

The weight and height of each subject were measured to the nearest 0.1 cm and 0.1 kg, respectively, and pubertal status was assessed according to standard Tanner's staging (27). During the initial assessment 85 girls were prepubertal. Six PA girls presented with Tanner stage II breast development. Central precocious puberty was excluded in all these cases by prepubertal responses to the Luteinizing Hormone Releasing Hormone (LHRH) stimulation test, low serum estradiol concentrations and prepuberal sonographic findings on pelvic ultrasound, indicating isolated premature thelarche.

Body mass index (BMI) was calculated by dividing body weight in kilograms by height in meters squared. Waist circumference (WC) and hip circumference (HC) were assessed in standing position by using a non-stretched and flexible tape. WC was measured midway between the lowest border of rib cage and the upper border of iliac crest, at the end of normal expiration. HC was measured at the widest part of the hip at the level of the greater trochanter (28). Waist-to-hip ratio (WHR) was calculated by diving waist to hip circumference and waist-to-height ratio (WHtR) by dividing waist circumference to height. Blood pressure was measured using an automated sphygmomanometer after the girls had rested for 5 min in sitting position. Neck and thyroid examination and all clinical assessments were performed by the same physician (EMK).

### Study protocol-endocrine and biochemical assessment-assays

Baseline blood samples were obtained between 8:00 and 9:00 AM by venipuncture after 12 h overnight fasting in supine position. All samples were immediately centrifuged, and serum and plasma were separated and frozen at −80°C until assayed. **Basal levels of the following biochemical and hormonal parameters were measured: (i) glucose, hemoglobin A1c (HbA1c), total cholesterol, low-density lipoprotein cholesterol (LDL), high-density lipoprotein cholesterol (HDL), triglycerides (ii) serum high-sensitivity C-reactive protein (hs-CRP) was quantitatively measured using a latex-enhanced immunoturbidimetric assay on a Roche Cobas 6,000 clinical chemistry analyzer. The detectable limit was 0.01 mg/dl, and the interassay and intraassay coefficients of variation were <2% (iii) FT4, TSH, anti-TPO, anti-TG, IGF-1 (insulin-like growth factor 1), IGF binding protein 3 (IGFBP-3), adrenocorticotropic hormone (ACTH), DHEAS (dehydroepiandrosterone sulfate), *Δ*4-androstenedione (*Δ*4-*Α*), testosterone, sex hormone-binding globulin (SHBG), estradiol, luteinizing hormone (LH), follicle stimulating hormone (FSH) and prolactin were measured using validated chemiluminescence immunoassays (CLIA) on the Immulite 2,000 automated analyser (Siemens Llanberis, Gwynedd LL55 4EL, United Kingdom). Quantification of cortisol, insulin and total 25-hydroxyvitamin D [25 (OH)D] was performed based on electrochemiluminescence immunoassay principle (ECLIA) of Cobas e411 automated analyser (Roche Diagnostcs GmbH, Mannheim, Germany). Serum 17-OHP concentrations were measured using ELISA (DIAsource Immunoassays, Ottignies-Louvain-la-Neuve, Belgium). Concentration of IGF binding protein 1 (IGFBP-1) was measured using validated ELISA (Mediagnost, Reutlingen, Germany) with a reported sensitivity of 0.055 ng/ml and total precision CV < 6.5% (assay range 0.1–8 ng/ml). Circulating levels of leptin and adiponectin were detected by validated ELISA kits (BioVendor, Brno, Czech Republic). Analytical sensitivity for leptin is 0.2 ng/ml (assay range 1–50 ng/ml) and for adiponectin 26 ng/ml (assay range 0.1-10 *μ*g/ml). Total precision CV is <8.0% for both ELISA kits. Concentrations of tumor necrosis factor-alpha (TNF-α) were determined using the high-sensitivity enzyme-linked immunosorbent assay (ELISA, Invitrogen, Bender MedSystems GmbH, Vienna, Austria), with a lower detection limit of 0.13 pg/ml and total precision coefficient of variation (CV) < 9,8%, (assay range 0.31-20 pg/ml).**

All girls with PA underwent a standard dose Synachten Stimulation test (SDSST) as well as an oral glucose tolerance test (OGTT), on two different days, between 8:00 and 9:00 AM after 12 hr overnight fasting. The SDSST was carried out first. 250 *μ*g of cosyntropin (synthetic ACTH) were administered intravenously and cortisol and 17-OHP were measured at baseline, 30 and 60 min after administering cosyntropin. An OGTT was performed by administering 1.75 g glucose/kg body weight (max 75 g) to each participant and blood samples for glucose and insulin measurements were drawn at baseline, 30 min, 60 min, 90 min and 120 min. Homeostasis model assessment of insulin resistance (HOMA-IR) and the quantitative insulin-sensitivity check index (QUICKI) were calculated as follows: HOMA-IR = [fasting glucose (mmol/l)×fasting insulin (*μ*U/ml)/22.5] and QUICKI = 1/Log (fasting Insulin, µU/ml) + Log (fasting glucose, mg/dl) (29).

An x-ray of the left hand and wrist was obtained in all PA subjects and bone age (BA) was determined using the method of Greulich and Pyle (30).

A thyroid ultrasound was performed in case of goiter, hypothyroidism, and in all subjects with AT**, by the same pediatric radiologist familiar with thyroid disease applying standard diagnostic criteria for AT and using the same device.**

### Statistical analysis

Categorical variables are presented as absolute and relative (%) frequencies. Quantitative variables are presented as median (min, max). In terms of graphical representation, quantitative variables are presented with boxplots, when needed. To compare quantitative variables between groups Kruskal-Wallis test was employed, while for pairwise comparisons Mann-Whitney test was used. Linear regression was used to investigate whether hormonal levels differed among the four groups when adjusting for the effect of BMI. Finally, Spearman's correlation coefficient (*ρ*) was used to investigate linear associations between quantitative variables. A 2-tailed *p*-value < 0.05 was considered statistically significant. Statistical analysis was implemented using *ΙΒΜ* SPSS v. 26 (IBM Corp. Released 2019. IBM SPSS Statistics for Windows, Version 26.0. Armonk, NY: IBM Corp.).

### Ethics

The study protocol was approved by the Ethics Committee of the “Aghia Sophia” Children's Hospital, Athens. Written informed consent was obtained from children's parents, and assent was obtained from the children before children have been enrolled in the study.

## Results

Among 73 girls with PA, 19 had AT (26%). Of these 19 girls with PA and concomitant AT, 6 were positive for both thyroid autoantibodies, 6 for anti-TPO only, and 3 for anti-TG only, whereas 4 were negative for both antibodies. Those 4 girls had already been diagnosed with subclinical hypothyroidism, were euthyroid under levothyroxine treatment and showed ultrasonographic findings reminiscent of AT when evaluated in our Department. Overall, the thyroid ultrasound showed typical sonographic features of AT in 13/19 girls. Extrathyroidal autoimmune diseases were present in 2/19 subjects: 1 had psoriasis and 1 celiac disease.

Based on the presence of AT and/or PA our whole study population was divided in 4 groups: Group PA-/AT+ consisted of those 6 girls who had AT without PA; Group PA+/AT− included 54 (out of 73) PA subjects without AT, **among whom: 2 with a history of transient congenital hypothyroidism and 2 with subclinical hypothyroidism under levothyroxine substitution treatment;** the above mentioned 19 (of 73) girls with PA and concomitant AT **formed** Group PA+/AT+ **including those 4 girls with subclinical hypothyroidism, under levothyroxine replacement therapy and sonographic features of AT;** and 12 healthy girls referred for investigation of growth with neither AT nor PA formed Group PA−/AT− of our study (control group).

### Clinical and biochemical parameters

**Figure 1:**
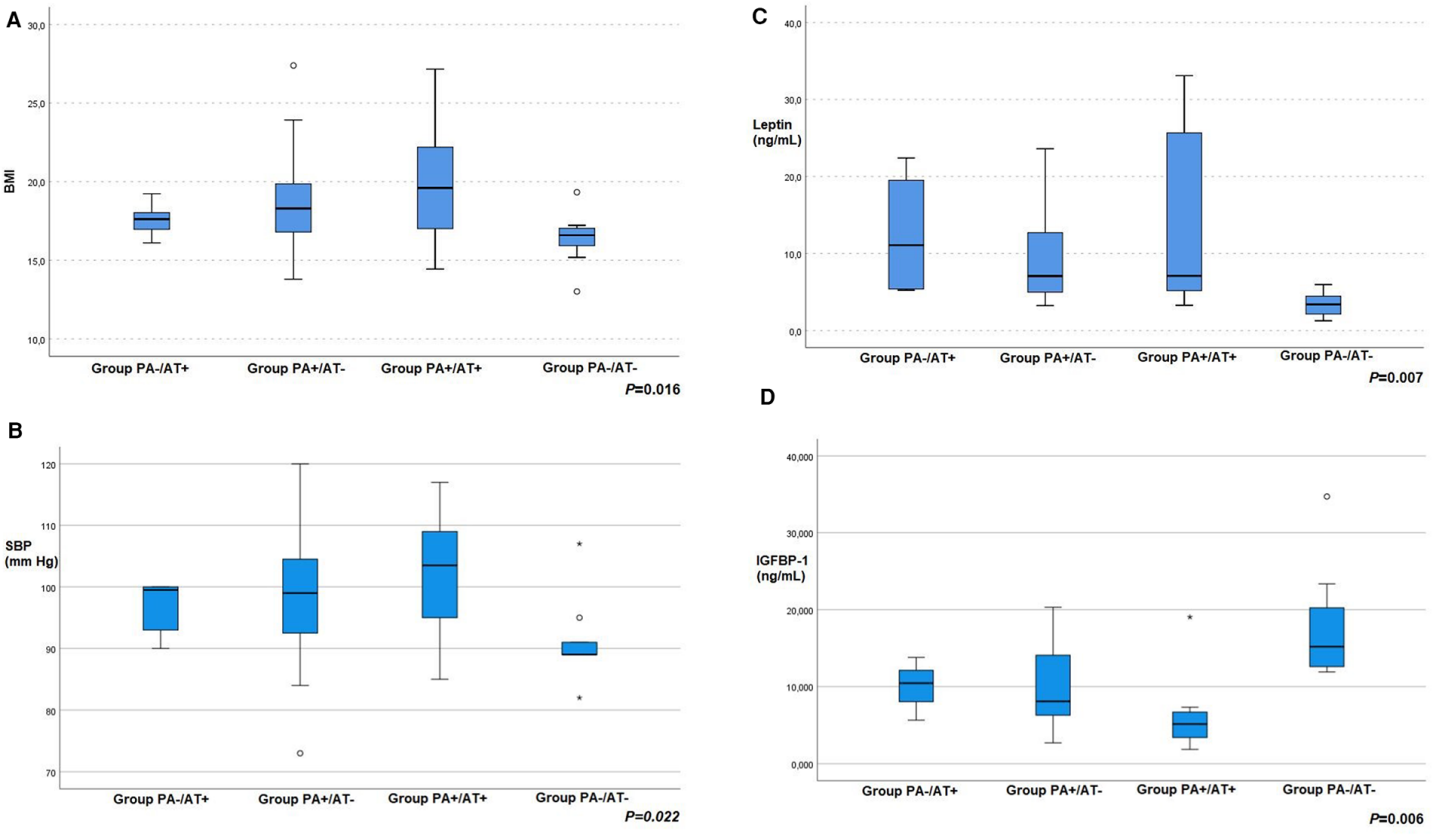
(**A**) body mass index (BMI) (**B**) systolic blood pressure (SBP) (**C**) leptin and (**D**) insulin-like growth factor-binding protein-1 (IGFBP-1) values of girls with autoimmune thyroiditis (AT) but no premature adrenarche (PA) (group PA−/AT+); girls with PA but no AT (group PA+/AT−); girls with PA and AT (group PA+/AT+); and girls without PA or AT [group PA−/AT− (controls)].

The participants of the four groups did not differ in age, birthweight, gestational age, mode of conception or delivery, family history for thyroid disorders, obesity, diabetes, PCOS, dyslipidemia, or cardiovascular disease. In terms of anthropometric and clinical variables, BMI was significantly different between the four groups (*p* = 0.016) (Figure 1A). Furthermore, a significant difference was observed in the frequency of goiter (*p* < 0.001), i.e., 33.3% in group PA-/AT+ (2/6), 1.85% in PA+/AT− (1/54) and 42.1% in PA+/AT+ (8/19), whereas none of the control girls had a goiter (0/12). No difference was observed with regards to WHR or WHtR.

Considering cardiovascular parameters systolic blood pressure was significantly different between groups (*p* = 0.022) (Figure 1B). No statistical important differences were detected in glucose, HbA1c, lipid profile and hsCRP levels. The clinical and laboratory characteristics of all participants are shown in Tables 1, 2, respectively.

**Table 1:** Clinical characteristics of girls in all study groups.

	Group PA-/AT+ (*n* = 6)	Group PA+/AT- (*n* = 54)	Group PA+/AT+ (*n* = 19)	Group PA-/AT- (*n* = 12)	*P* value
**Age**	6.25 (5.83–9.58)	7.25 (4.00–8.75)	7.83 (5.42–10.08)	7.13 (5.50–8.75)	0.161
**Gestational age (weeks)**	40.00 (37.71–40.86)	38.00 (28.00–40.14)	38.00 (32.57–40.00)	37.30 (34.29–41.00)	0.218
**Birth weight (g)**	3325 (2600–3670)	2975 (1275–4200)	2850 (1550–4140)	2800 (1730–4200)	0.201
**BMI (kg/m^2^)**	17.62 (16.10–19.23)	18.30 (13.79–27.39)	19.60 (14.44–27.16)	16.59 (13.01–19.33)	**0**.**016**
**WHR**	0.90 (0.88–0.91)	0.84 (0.76–0.98)	0.86 (0.79–0.93)	0.89 (0.81–0.95)	0.310
**WHtR**	0.50 (0.48–0.51)	0.45 (0.39–0.64)	0.45 (0.41–0.59)	0.45 (0.41–0.48)	0.302
**Systolic BP**	99.5 (90.0–100.0)	99.0 (73.0–120.0)	103.5 (85.0–117.0)	89.0 (82.0–107.0)	**0**.**022**
**Diastolic BP**	59.5 (52.0–68.0)	63.0 (41.0–78.0)	67.0 (41.0–77.0)	57.0 (49.0–66.0)	0.053
**Goiter, *n* (%)**	2/6 33.3	1/54 18.5	8/19 42.1%	0/12 0.0	**<0** **.** **001**

Group PA−/AT+, girls with autoimmune thyroiditis (AT) but no premature adrenarche (PA); Group PA+/AT−, girls with PA but no AT; Group PA+/AT+, girls with PA and AT; Group PA−/AT−, girls without PA or AT (controls); *n* = number; g, gram; BMI, body mass index; kg, kilograms; m^2^, meters squared; WHR, waist-to-hip ratio; WHtR, waist-to-height ratio; BP, blood pressure; the results were presented as the median values (min, max). Comparisons between groups were performed using Kruskal-Wallis test.

**Table 2:** Metabolic and endocrine parameters of girls in all study groups.

	Group PA− /AT+(*n* = 6)	Group PA+/AT− (*n* = 54)	Group PA+/AT+ (*n* = 19)	Group PA−/AT− (*n* = 12)	*P* value
Glucose (mg/dl)	81.5 (74.0–89.0)	82.5 (68.0-106.0)	82.5 (67.0-99.0)	78.0 (69.0-104.0)	0.635
HbA1c (%)	5.20 (4.90–5.70)	5.40 (4.70-5.90)	5.35 (4.90-5.80)	5.20 (5.00-5.50)	0.087
Total Cholesterol (ng/dl)	177 (154–205)	163 (119-207)	169 (115-212)	160 (120-193)	0.137
LDL (mg/dl)	106 (90–114)	88 (40-142)	96 (57-134)	90 (52-121)	0.125
HDL (mg/dl)	59 (48–88)	61 (42-79)	55 (42-99)	62 (42-78)	0.579
Triglycerides (mg/dl)	54 (43–154)	53 (33-136)	62 (37-123)	49 (25-133)	0.664
hs-CRP (mg/l)	2.19 (1.00–3.24)	1.14 (1.00-3.93)	1.65 (1.00-3.94)	1.20 (0.00-1.57)	0.140
FT4 (ng/dl)	1.32 (1.08–1.52)	1.17 (0.86-1.65)	1.21 (0.96-2.36)	1.12 (0.99-1.34)	0.051
TSH (μUI/ml)	3.28 (1.24–4.23)	2.47 (0.71-4.78)	3.09 (0.84-5.00)	1.82 (1.21-3.90)	**0**.**044**
Anti-TPO (IU/ml)	30.59 (10.00–173.00)	10.00 (0.22-32.60)	39.29 (1.00-982.00)	10.00 (10.00-23.30)	**0**.**002**
Anti-TG (IU/ml)	20.00 (12.70–1386.0)	20.00 (1.40-20.80)	58.00 (2.20-3000.0)	20.00 (20.00-20.00)	**0**.**044**
25 (OH)D (ng/ml)	21.90 (19.19–36.00)	24.90 (11.50-41.80)	21.95 (7.30-37.00)	28.25 (20.00-42.00)	0.143
Insulin (μUI/ml)	6.67 (6.38–9.34)	8.40 (1.30-24.61)	8.00 (4.50-21.38)	8.10 (5.06-15.60)	0.861
HOMA	1.40 (0.10–1.80)	1.80 (0.20-5.80)	1.65 (1.00-6.50)	1.70 (0.90-4.00)	0.655
QUICKI	0.36 (0.35–0.36)	0.35 (0.30-0.51)	0.35 (0.29-0.39)	0.35 (0.31-0.39)	0.891
IGF-1 (ng/ml)	156.5 (124.0–196.0)	195.0 (103.0-342.0)	165.0 (110.0-374.0)	149.0 (70.1-284.0)	**0**.**012**
IGFBP-3 (ng/ml)	5.25 (4.09–5.58)	4.98 (3.03-6.71)	5.23 (3.65-7.59)	4.25 (3.27-5.11)	**0**.**049**
IGFBP-1 (ng/ml)	10.45 (5.65–13.79)	8.08 (2.69-20.32)	5.14 (1.84-19.04)	15.19 (11.90-34.72)	**0**.**006**
Cortisol (μg/dl)	15.87 (10.70–20.50)	11.74 (4.98-22.99)	14.56 (3.94-29.10)	10.60 (6.86-27.80)	0.442
ACTH (pg/ml)	25.22 (19.30–29.80)	18.59 (7.64-59.60)	27.56 (11.50-37.82)	19.22 (7.36-87.70)	0.902
17-OHP (ng/ml)	0.60 (0.26–1.04)	0.79 (0.22-3.14)	0.72 (0.40-1.45)	0.51 (0.21-1.77)	0.058
DHEA-S (μg/dl)	20.10 (5.00–117.00)	66.55 (12.20-202.00)	60.50 (15.00-162.00)	21.35 (0.44-42.10)	**<0** **.** **001**
*Δ*4-*Α* (ng/ml)	0.30 (0.06–0.52)	0.40 (0.06-1.61)	0.40 (0.15-1.38)	0.30 (0.24-0.39)	**0**.**010**
Testosterone (ng/dl)	10.00 (0.20–20.00)	20.00 (0.20-25.20)	20.00 (0.30-21.00)	20.00 (0.20-20.00)	0.261
SHBG (nmol/l)	109.00 (83.61–119.00)	61.36 (31.71-193.70)	66.79 (22.50-133.90)	112.05 (45.90-179.00)	0.177
Leptin (ng/ml)	11.08 (5.23–22.40	7.08 (3.24-23.60)	7.10 (3.28-33.10)	3.39 (1.26-5.96)	**0**.**007**
Adiponectin (μg/ml))	13.55 (10.40–16.10)	11.75 (5.96–20.70)	10.20 (7.67-11.40)	10.60 (8.29-13.80)	0.233
TNF-α (pg/ml)	0.80 (0.16–5.54)	0.83 (0.19–1.28)	0.85 (0.47-1.20)	0.92 (0.00-2.04)	0.987

Group PA−/AT+, girls with autoimmune thyroiditis (AT) but no premature adrenarche (PA); Group PA+/AT−, girls with PA but no AT; Group PA+/AT+, girls with PA and AT; Group PA−/AT−, girls without PA or AT (controls). Comparisons between groups were performed using Kruskal-Wallis test.

Reference ranges: Glucose: 70-100 mg/dl, Hemoglobin A1c (HbA1c) < 6.1%; total cholesterol: 120-200 mg/dl; low-density lipoprotein cholesterol (LDL): < 130 mg/dl;, high-density lipoprotein cholesterol (HDL): 35–84 mg/dl; triglycerides: 30–130 mg/dl; high-sensitivity C-reactive protein (hs-CRP): < 2 mg/l; free thyroxine (FT4): 0.65–1.78 ng/dl; thyroid-stimulating hormone (TSH): 0.5-5 μUI/ml; anti–thyroid peroxidase antibody (anti-TPO): 0–35 U/ml; anti-thyroglobulin antibody (anti-TG): 0–40 U/ml; 25-hydroxyvitamin D [25 (OH)D]: 20–80 ng/ml; insulin: 2.6-25 μUI/ml; IGF-1 (insulin-like growth factor 1): 57–277 ng/ml 7–9 years old, 71-394 ng/ml Tanner stage I; IGF binding protein 3 (IGFBP-3): 1.4–6.1 μg/ml, IGF binding protein 1 (IGF-BP1): 0.1–8 ng/ml; cortisol: 5–25 μg/dl; adrenocorticotropic hormone (ACTH): 9–52 pg/ml; 17-hydroxyprogesterone (17-OHP): 0.15–0.65 ng/ml 1-9 years old, 0.2–0.8 ng/ml 9–11 years old; DHEAS (dehydroepiandrosterone sulfate): 6–77 μg/dl 4–9 years old, 25–214 μg/dl 9-13 years old; *Δ*4-androstenedione (*Δ*4-*Α*): 0.05–0.45 ng/ml 2-9 years old, 0.25–0.8 ng/ml 9–11 years old; testosterone: 1–20 ng/dl 1–10 years old; sex hormone-binding globulin (SHBG): 18–114 nmol/L, prolactin (PRL): 5–20 ng/ml; leptin: 1–50 ng/ml, adiponectin: 0.1–10 μg/ml; tumor necrosis factor-alpha (TNF-α): 0.31–20 pg/ml; results are presented as the median values (min, max).

### Hormonal features

The endocrine features of all study groups are summarized in Table 2. When comparing hormonal parameters among the four groups of our study significant differences were found in leptin (*p* = 0.007), TSH (*p* = 0.044), anti-TPO (*p* = 0.002), anti-TG (*p* = 0.044), IGF-BP1 (*p* = 0.006), *Δ*4-*Α* (*p* = 0.01), DHEA-S (*p *= <0.001), IGF-1 (*p* = 0.012) and IGF-BP3 (*p* = 0.049) levels (Figures 1C, D]. In particular, TSH levels were significantly higher in Group PA+/AT+ compared to Groups PA+/AT− and PA−/AT− (*p* = 0.043 and *p* = 0.016, respectively). Moreover, girls with AT (Groups PA−/AT+ and PA+/AT+) had higher TSH levels than girls in Group PA+/AT− (*p* = 0.025). In addition, after adjustment for BMI, leptin concentrations were significantly higher in Group PA-/AT+ compared to Group PA-/AT− (*p* =  0.048) (Table 3). No differences were noted in TNF-α or adiponectin levels between groups. There were no differences with respect to insulin resistance indices, i.e., HOMA-IR and QUICKI between the four groups.

**Table 3:** Comparison of anthropometric and hormonal data between girls of group PA+/AT+ and those of groups PA−/AT−, PA−/AT+ and PA+/AT−.

Variable	Group PA+/AT + vs. Group PA−/AT−	Group PA+/AT + vs. Group PA−/AT+	Group PA+/AT + vs. Group PA+/AT−
	*P* value		
BMI	**0**.**012**	0.279	0.261
Leptin	**0**.026*	0.788	0.542
TSH	**0**.**016**	0.955	**0**.**043**
FT4	0.056	0.529	0.082

Group PA−/AT+, girls with autoimmune thyroiditis (AT) but no premature adrenarche (PA); Group PA+/AT−, girls with PA but no AT; Group PA+/AT+, girls with PA and AT; Group PA−/AT−, girls without PA or AT (controls).

Vs: vs.; BMI: body mass index; TSH: thyroid-stimulating hormone; FT4: free thyroxine.

* We used linear regression to investigate whether leptin levels differed among the four groups when adjusting for the effect of BMI. Group PA+/AT+ did not differ significantly from any other group, when adjusting for BMI. The only statistically significant difference after adjusting for BMI was observed between Groups PA−/AT+ and PA−/AT− (*p* = 0.048). All other pairwise comparisons were not statistically significant.

**Figure 2:**
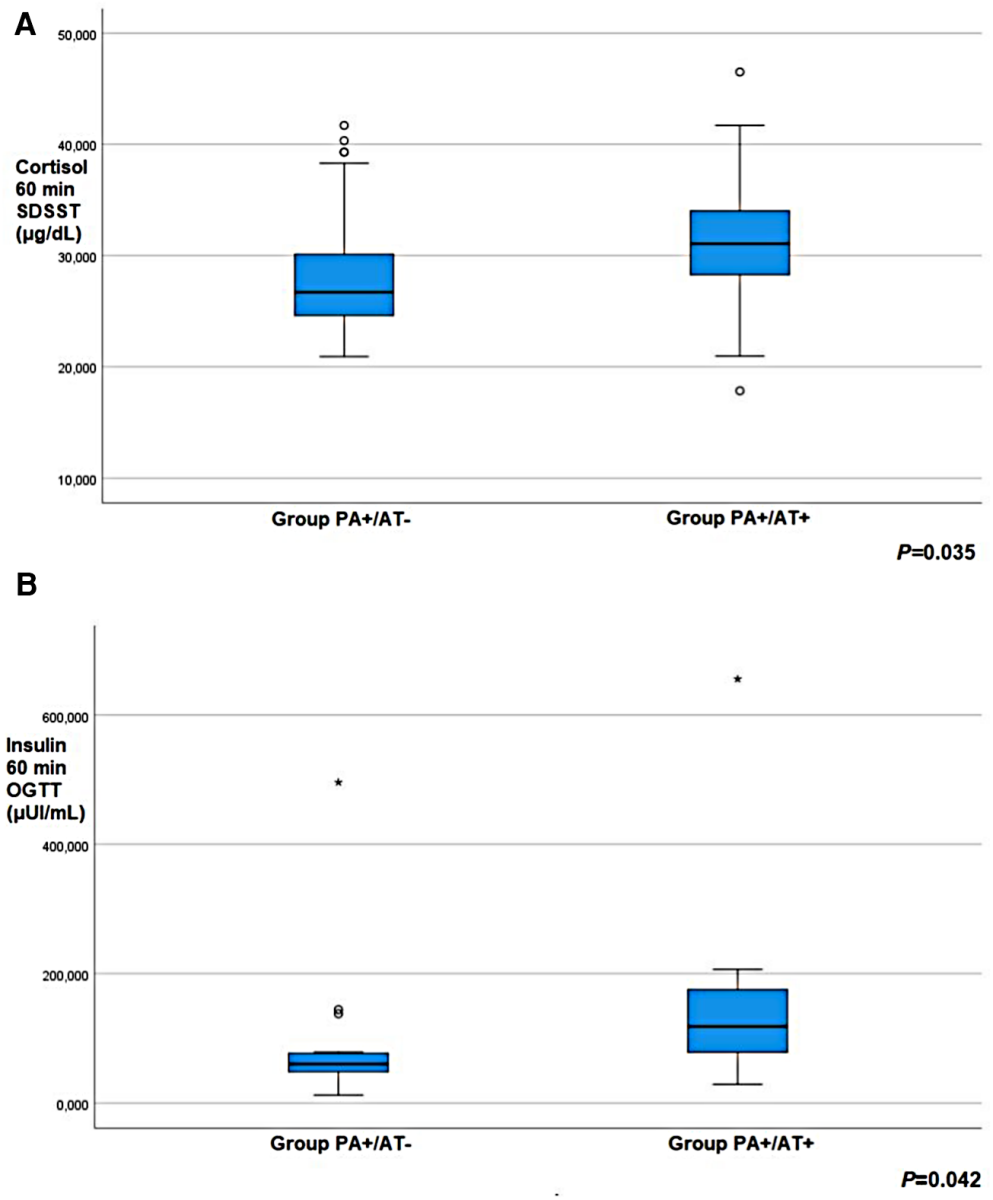
(**A**) cortisol response at 60 min post-standard dose synacthen stimulation test (SDSST) and (**B**) insulin concentrations at 60 min during oral glucose tolerance test (OGTT) in girls with premature adrenarche (PA) but no autoimmune thyroiditis (AT) (group PA+/AT−); girls with PA and AT (group PA+/AT+). Small circles denote outlier values; asterisks denote extreme outlier values.

Girls in Group PA+/AT + showed higher cortisol response at 60 min post-SDSST than girls in Group PA+/AT- (*p* = 0.035) (Table 4, Figure 2A). During OGTT, insulin concentrations at 60 min after glucose load were significantly higher in Group **PA+/AT+** compared to Group PA+/AT− (*p* = 0.042) (Table 4, Figure 2B). The difference between the bone age and the chronological age was similar in Groups PA+/AT− and PA+/AT + .

**Table 4:** Comparison of a) cortisol and 17-OHP b) glucose and insulin responses to SDSST and OGTT, respectively, between groups PA+/AT− and PA+/AT+.

SDSST			
	Groups	Median	*P* value
Cortisol 0 min (μg/dl)	Group PA+/AT−	11.74 (4.69–26.90)	0.329
	Group PA+/AT+	14.76 (3.58–29.10)	
Cortisol 30 min (μg/dl)	Group PA+/AT−	23.90 (15.83–35.60)	0.060
	Group PA+/AT+	26.22 (18.80–39.84)	
Cortisol 60 min (μg/dl)	Group PA+/AT−	26.71 (20.92–41.70)	**0**.**035**
	Group PA+/AT+	31.06 (17.85–46.51)	
17-OHP 0 min (ng/ml)	Group PA+/AT−	0.90 (0.22–2.16)	0.971
	Group PA+/AT+	0.85 (0.48–2.56)	
**17-OHP** 30 min (ng/ml)	Group PA+/AT−	2.04 (0.88–3.39)	0.993
	Group PA+/AT+	2.05 (1.28–3.53)	
**17-OHP** 60 min (ng/ml)	Group PA+/AT−	2.23 (0.27–3.95)	0.693
	Group PA+/AT+	2.36 (1.36–3.99)	
**OGTT**
Glucose 0 min (mg/dl)	Group PA+/AT−	85.00 (81.00–93.00)	0.472
	Group PA+/AT+	88.00 (73.00–96.00)	
Glucose 30 min (mg/dl)	Group PA+/AT−	152.00 (79.00–214.00)	0.144
	Group PA+/AT+	168.50 (101.00–175.00)	
Glucose 60 min (mg/dl)	Group PA+/AT−	126.00 (77.00–209.00)	0.123
	Group PA+/AT+	151.00 (97.00–179.00)	
Glucose 90 min (mg/dl)	Group PA+/AT−	117.00 (83.00–217.00)	0.458
	Group PA+/AT+	118.00 (103.00–149.00)	
Glucose 120 min (mg/dl)	Group PA+/AT−	107.00 (73.00–135.00)	0.537
	Group PA+/AT+	108.50 (85.00–136.00)	
Insulin 0 min (μUI/ml)	Group PA+/AT−	8.80 (4.92–24.96)	0.164
	Group PA+/AT+	12.79 (7.08–31.11)	
Insulin 30 min (μUI/ml)	Group PA+/AT−	75.82 (31.34–245.60)	0.210
	Group PA+/AT+	100.35 (49.52–439.40)	
Insulin 60 min (μUI/ml)	Group PA+/AT−	60.41 (12.22–495.80)	**0**.**042**
	Group PA+/AT+	118.35 (28.94–655.70)	
Insulin 90 min (μUI/ml)	Group PA+/AT−	56.25 (13.90–397.30)	0.057
	Group PA+/AT+	101.33 (42.10–376.10)	
Insulin 120 min (μUI/ml)	Group PA+/AT−	40.31 (18.45–200.90)	0.091
	Group PA+/AT+	66.88 (32.72–451.60)	

Group PA+/AT−, girls with PA but no AT; Group PA+/AT+, girls with PA and AT; SDSST, standard dose **synachten** stimulation test; **17-OHP, 17-hydroxyprogesterone;** OGTT, oral glucose tolerance test; the results were expressed as the median values (min, max). Comparisons between groups were performed using Mann-Whitney test.

Finally, in the entire cohort (*n* = 91) the following significant associations were found: BMI correlated positively to TSH (*ρ *= 0.243, *p* = 0.021), DHEA-S (*ρ *= 0.320, *p* = 0.003) and **17-OHP** (*ρ *= 0.242, *p* = 0.022); TSH positively correlated to *Δ*4-*Α* (*ρ *= 0.227, *p* = 0.04) and **17-OHP** (*ρ *= 0.443, *p* < 0.001); insulin positively correlated to testosterone (*ρ *= 0.277, *p* = 0.027) and **17-OHP** (*ρ *= 0.251, *p* = 0.044); leptin positively correlated to **17-OHP** (*ρ *= 0.329, *p* = 0.038); IGF-BP3 to *Δ*4-A (*ρ *= 0.326, *p* = 0.031); and systolic blood pressure to **17-OHP** (*ρ *= 0.241, *p* = 0.037). On the other hand, FT4 inversely correlated to testosterone (*ρ *= −0.364, *p* = 0.001) and SHBG inversely correlated to DHEA-S (*ρ *= −0.334, *p* = 0.029) and *Δ*4-*Α* (*ρ *= −0.407, *p* = 0.007).

## Discussion

The association between AT and PCOS was first described more than a decade ago by Janssen et al., who identified AT in 26.9% of PCOS patients compared to 8.4% of controls (31). This almost threefold higher prevalence of AT in PCOS patients was subsequently confirmed by other investigators (11, 15, 16, 32–35). On the other hand, a higher prevalence of PCOS (46.8%) compared to controls (4.3%) was observed among 13–18-year-old euthyroid girls with AT (14), suggesting a relationship between these two conditions (36). The clinical features, hormonal and metabolic derangements of PCOS may emerge during childhood, first expressed as PA and evolving later to PCOS (17). Previous studies have shown an increased frequency of insulin resistance, cardiometabolic aberrations and ovarian hyperandrogenism in girls with a history of PA (21–23, 37–39), indicating that PA may be a harbinger of PCOS rather than a normal variant. In this regard, the risk of developing PCOS in patients with premature pubarche seems to be 15%–20% (17).

The present study revealed a high frequency of AT (i.e 26%) among euthyroid prepubertal girls with PA that is significantly higher than the one reported in healthy schoolgirls in our country, being 2.2% in prepubertal and 8.2% in pubertal girls, respectively (40). In concordance with studies from other countries, the prevalence of **AT** in the pediatric age has been reported to range between 1.2%–9.6%, reaching a peak in early to mid-adolescence (7). As expected (40, 41), the presence of goiter in our study was more frequent in girls with positive thyroid autoantibodies (with or without PA).

To the best of our knowledge, this is the first study to investigate an association between PA and thyroid autoimmunity in euthyroid prepubertal girls. In relation to our findings, three possible mechanisms linking PA and AT could be speculated: i) obesity ii) insulin resistance iii) increased leptin levels and associated immune dysregulation (36).

With respect to the role of obesity, our analysis demonstrated significant differences in BMI values among the four groups of our study. Of interest, there was a trend -although not statistically significant- towards higher BMI in girls with PA and concomitant AT.

The association between increased BMI and both PA-PCOS is well documented (13, 19). Moreover, an intriguing link between adiposity and AT has been increasingly reported. Overall, the frequency of AT in obese subjects ranges between 5.6%–12.4% in children and 10%–23.6% in adults (10, 42–45). In the United Kingdom Medical Research Council 1946 British Birth Cohort study, levothyroxine use and positive anti-TPO antibodies in women at age 60–64 years were positively associated with body weight or overweight in childhood and adult BMI. Furthermore, women who were overweight or obese at age 14 years had a higher risk of developing positive anti-TPO antibodies later in life (46).

**Of interest, patients with both PCOS and AT were more obese by 2 kg/m² BMI on average than PCOS subjects without AT** (**16**)**.**

Similarly, euthyroid girls with AT and PCOS had higher BMI, fasting glucose, cholesterol and HOMA-IR compared to girls with **AT** alone or controls (14).

The pathophysiological link between obesity and AT remains largely unclear, however growing evidence points to reduced immunological tolerance secondary to an altered balance of adipokines (e.g., leptin and adiponectin) and cytokines [e.g., interleukin 6 and TNF-α) produced by the white adipose tissue and resultant abnormalities of the immune response (47).

In addition, numerous studies have demonstrated higher TSH values in overweight or obese compared to normal-weight subjects (48, 49). BMI was positively correlated with TSH and negatively with serum fT4 levels in both adults and children (50, 51), suggesting that mild variations in thyroid function even within the reference range may influence the regulation of body weight or, inversely, may be influenced by the body weight (51).

On the other hand, increased TSH levels were more frequently observed in children with high anti-TPO titers. A positive correlation between TSH and anti-TPO levels has been previously reported, indicating that AT could be linked to an increased prevalence of subclinical hypothyroidism (10, 40, 41, 49, 52).

Our findings, i.e., the significant positive correlation between TSH and BMI in the whole study population and the higher TSH levels in AT girls with or without PA are in agreement with these findings, suggesting a possible link between thyroid hormonal status, obesity and thyroid autoimmunity.

Hyperthyrotropinaemia and a moderate increase in thyroid hormone concentrations have been observed in obese children, but these are mostly considered as an adaptation process to weight gain, being the consequence of overweight rather than the cause of it. Nevertheless, aberrations in thyroid function usually normalize after moderate weight loss, indicating that they may be reversible (44, 53). The pathophysiology underlying these alterations remains **unclear, however, several mechanisms have been proposed, including: 1)** an impaired negative feedback mechanism, due to a reduced number of T3 receptors in the pituitary **in obese patients (pituitary resistance)** 2) peripheral resistance to thyroid hormones associated with reduced expression of thyroid genes, particularly TSHR, in subcutaneous and visceral fat **in obese subjects** 3) obesity-induced chronic low-grade inflammation, characterized by release of inflammatory cytokines from the adipose tissue, such as TNF-α, interleukin-1 and interleukin-6 which were shown to have an inhibitory effect on sodium/iodide symporter (NIS) **mRNA expression, thus possibly contributing to compensatory TSH elevation** 4) abnormal activity of the deiodinase enzymes D1 and D2 in adiposity resulting in a higher FT3 to FT4 ratio 5) development of thyroid autoimmunity 6) increased leptin levels promoting TRH secretion from the hypothalamus (42, 44–46, 53).

With regards to this latter mechanism, and in concordance with previous studies we found higher leptin levels in girls with AT compared to controls (43, 54). Leptin is a 16-kD adipokine released from the white adipose tissue in proportion to body fat mass, with rising levels as BMI increases (55, 56). Leptin has pleiotropic effects, regulating appetite and energy expenditure but also neuroendocrine function, metabolism, and immune responses (56, 57). Leptin activates pro-thyrotropin releasing hormone (pro-TRH) expression, which results in TRH release and increased TSH secretion (53). Moreover, leptin receptor is expressed in normal CD4+, CD8+ T cells, NK cells and B cells, mediating the immunomodulatory actions of leptin, which has been shown to enhance the Th1 response (43, 57–59). **Among obese subjects, those with AT had higher leptin levels. Of note, leptin levels were associated with AT, independent of fat mass and BMI** (**43**)**. On the other hand, a subsequent study demonstrated a positive correlation between increased leptin levels and thyroid autoantibodies in nonobese males** (**56**).

Finally, insulin resistance has been increasingly recognized as contributing factor to the development of thyroid autoimmunity. Higher fasting glucose, insulin and HOMA-IR values were observed in euthyroid AT individuals (8, 60). TSH levels were found to positively correlate with insulin and HOMA-IR levels (8, 61). Moreover, anti-TPO titers were positively associated to HOMA-IR and hsCRP levels, independently of thyroid function in non-obese subjects, indicating that mild changes of thyroid function even in the euthyroid state, chronic inflammation, and insulin resistance may be implicated in the development of AT (8, 9). On top of the above, patients with PCOS and concomitant AT showed higher total cholesterol, TSH, HOMA-IR and insulin levels at both 30 and 60 min post-OGTT, lower FT4 and FT4/TSH ratio compared to those without AT (62). Furthermore, as previously reported in the study of Ganie et al., euthyroid girls with HT and PCOS had higher fasting glucose, cholesterol and HOMA-IR compared to girls with HT alone or controls. In addition, there was a significant inverse correlation between serum FT4 quartiles and various components of PCOS (14). The above findings imply that the concurrence of AT and PCOS may exacerbate the metabolic aberrations observed in PCOS or AT alone. In line with these observations, we found higher insulin concentrations at 60 min during OGTT in girls with PA and AT compared to those with PA only. Hyperinsulinemia and insulin resistance associated with variable components of the metabolic syndrome are well known features in girls with PA (21–23, 37, 63, 64). Taking the above data into account it could be postulated that the combined occurrence of AT and PA may be associated with more pronounced metabolic abnormalities than observed in PA alone.

Another interesting finding of the present study was the higher cortisol response at 60 min post-SDSST in girls with both PA and AT than in those with PA only. Elevated levels of salivary and plasma cortisol have been previously observed in PA girls and attributed to hyperactivation of the hypothalamus-pituitary-adrenal (HPA) axis. It has been hypothesized, that overactivation of the HPA axis (e.g., related to obesity or prenatal stress exposure) and the resultant chronic cortisol hypersecretion in susceptible individuals may lead to visceral fat accumulation, insulin resistance, release of pro-inflammatory cytokines in adipose tissue and activation of signaling pathways implicated in the pathogenesis of PCOS and cardiovascular disease later in life (20, 65, 66). The positive correlation between 17-OHP levels and systolic blood pressure in our study might indirectly reflect an association between chronic dysregulation of the HPA axis, resultant hypercortisolism and the development of components of the metabolic syndrome.

In addition, the higher cortisol together with higher insulin levels observed in PA girls with AT, points towards a worse metabolic and endocrine profile, which -through mechanisms already discussed- might lead to immune alterations and thus render these individuals more prone to the development of thyroid autoimmunity and/or PCOS later in life.

Given that most girls included in our study are girls with PA, the correlations observed between clinical and endocrine parameters reflect mainly abnormalities **frequently encountered in PA subjects** (21, 23, 37, 63, 64). **Interestingly, girls with PA and concomitant AT tended to have lower IGF-BP1 levels compared to the other groups. As the hepatic production of both SHBG and IGF-BP1 is downregulated by insulin, their concentrations have been suggested as useful markers of insulin resistance** (37). **The above finding** could indirectly reflect a higher degree of insulin resistance in these subjects and is in parallel with the greater response of insulin during OGTT noted in the same group. Reduced IGF-BP1 levels would result in higher unbound (free) IGF-1 concentrations and subsequently an increase in both adrenal and ovarian androgen secretion (66). Indeed, both insulin and IGF-1 were shown to enhance the ACTH-driven adrenal steroidogenesis, thus contributing to increased androgen secretion during **adrenarche** (18, 37). The positive correlation between BMI and both DHEAS and 17-OHP observed in our study population is in alignment with **previous observations**, suggesting an association between obesity and androgen excess that may further aggravate the metabolic dysfunction of affected **individuals** (13). **Overall, the associations found in this study are in concordance with previous reports, pointing** towards a link between weight gain, elevation of leptin levels, hyperinsulinemia and consequently increased adrenal androgen production.

As previously mentioned, not only, BMI but also TSH has been linked to insulin resistance **indices** (**67**)**.** It is noteworthy, **that, among women with PCOS,** the association between thyroid function and IR was shown to be independent of age and BMI (**68**), underlining the important role of thyroid hormones on regulation of insulin sensitivity. **A** negative association between FT4 and insulin resistance indices has been **previously reported** (61). In addition, FT4 quartiles were negatively correlated with various PCOS components in euthyroid girls with AT (14). **Our findings, i.e.,** the positive correlation between TSH and androgen levels, **and** negative between FT4 and testosterone, are in concordance with previous observations, implying a possible association between insulin resistance and thyroid function in our study population.

The main strength of the present study is that we included unselected and well-characterized girls with PA, AT, and controls, who were evaluated applying the same protocol. To our knowledge, this is the first study aiming to investigate the clinical and endocrine features of girls with PA and concurrent AT, although there are some limitations too that need to be addressed. Firstly, the number of girls with AT and controls is small. With regards to the group with AT, the small sample size reflects the rarity of this condition among prepubertal children. Concerning the control group, the study was conducted during the COVID-19 pandemic and the number of children referred for evaluation in the outpatient setting especially for normal variants of growth and puberty was significantly reduced. Secondly, our study population consisted only of girls, so that our results cannot be extrapolated to boys. Moreover, before study entry all participants were prepubertal with normal TSH and FT4 levels. Taking into consideration that the role of estrogens in the development of autoimmune diseases is well established, puberty is a state of relative “normal” insulin resistance, and hypothyroidism is also associated with insulin resistance we stratified only euthyroid prepubertal girls to reduce the impact of confounding factors on our results, such as subclinical or overt thyroid dysfunction and hormonal changes occurring during different pubertal stages (37, 40, 60). **On the other hand, considering the existing data supporting a possible positive effect of levothyroxine treatment on thyroid autoimmune activity, we acknowledge that our results might have been affected by administration of levothyroxine therapy in a minority of our participants** (**60**). **Furthermore, hormonal measurements in our study were carried our using immunoassay-based techniques, which are widely used in clinical and research settings, albeit known to have reduced specificity, when compared with the mass spectrometry methods**.

In conclusion, the present study revealed a high frequency of AT among euthyroid prepubertal girls with PA**, and while waiting for larger studies to confirm our findings, screening** PA **girls** for autoimmune thyroid disease **would be justifiable.** Girls with PA and concomitant AT had higher TSH levels, cortisol response post-SDSST and insulin levels during OGTT compared to those with PA alone. Our findings indicate that a combination of PA with AT even in the euthyroid state may be associated with a greater degree of insulin resistance, which might aggravate the metabolic abnormalities observed in PA alone. The importance of achieving and maintaining a normal weight should by stressed and lifestyle changes and regular exercise should be encouraged in all overweight and obese PA children with or without AT. Further studies are required to better elucidate the underlying pathophysiological mechanisms linking PA and AT.

## Data Availability

The original contributions presented in the study are included in the article/Supplementary Material, further inquiries can be directed to the corresponding author.

## References

[1] Franco JS Amaya-Amaya J Anaya JM. Thyroid disease and autoimmune diseases (2013). In: Anaya JM Shoenfeld Y Rojas-Villarraga A, editors. Autoimmunity: From bench to bedside. Bogota (Colombia): El Rosario University Press. Chapter 30. https://www.ncbi.nlm.nih.gov/books/NBK459466/[Accessed July 18, 2013]. 29087650

[2] Gopalakrishnan S Marwaha RK. Juvenile autoimmune thyroiditis. Journal of Pediatric Endocrinology and Metabolism. (2007) 20:961–70. 10.1515/JPEM.2007.20.9.961 18038704

[3] Hanley P Lord K Bauer AJ. Thyroid disorders in children and adolescents: a review. JAMA Pediatr. (2016) 170:1008–19. 10.1001/jamapediatrics.2016.0486 27571216

[4] Kyritsi EM Kanaka-Gantenbein C. Autoimmune thyroid disease in specific genetic syndromes in childhood and adolescence. Front Endocrinol (Lausanne). (2020) 11:543. 10.3389/fendo.2020.00543 32973676 PMC7466763

[5] Kakourou T Kanaka-Gantenbein C Papadopoulou A Kaloumenou E Chrousos GP. Increased prevalence of chronic autoimmune (Hashimoto's) thyroiditis in children and adolescents with vitiligo. J Am Acad Dermatol. (2005) 53:220–3. 10.1016/j.jaad.2005.03.032 16021113

[6] Cappa M Bizzarri C Crea F. Autoimmune thyroid diseases in children. J Thyroid Res. (2010) 2011:675703. 10.4061/2011/675703 21209713 PMC3010678

[7] Segni M. Disorders of the thyroid gland in infancy, childhood and adolescence. In: Feingold KR Anawalt B Boyce A editors. Endotext. South dartmouth (MA): MDText.com, Inc.; (2000). [Accessed March 18, 2017] 25905261

[8] Varim C Kaya T Varim P Nalbant A Vatan MB Yaylaci S Insulin resistance in the patients with euthyroid hashimoto thyroiditis. Biomed Res. (2017) 28:1543–7.

[9] Liu J Duan Y Fu J Wang G. Association between thyroid hormones, thyroid antibodies, and cardiometabolic factors in non-obese individuals with normal thyroid function. Front Endocrinol (Lausanne). (2018) 9:130. 10.3389/fendo.2018.00130 29674996 PMC5895644

[10] García-García E Vázquez-López MA García-Fuentes E Galera-Martínez R Gutiérrez-Repiso C García-Escobar I Thyroid function and thyroid autoimmunity in relation to weight Status and cardiovascular risk factors in children and adolescents: a population-based study. J Clin Res Pediatr Endocrinol. (2016) 8:157–62. 10.4274/jcrpe.2687 26761948 PMC5096470

[11] Zeber-Lubecka N Hennig EE. Genetic susceptibility to joint occurrence of polycystic ovary syndrome and Hashimoto's Thyroiditis: how far is our understanding? Front Immunol. (2021) 12:606620. 10.3389/fimmu.2021.606620 33746952 PMC7968419

[12] Azziz R Carmina E Chen Z Dunaif A Laven JS Legro RS Polycystic ovary syndrome. Nat Rev Dis Primer. (2016) 2:16057. 10.1038/nrdp.2016.57 27510637

[13] Randeva HS Tan BK Weickert MO Lois K Nestler JE Sattar N Cardiometabolic aspects of the polycystic ovary syndrome. Endocr Rev. (2012) 33:812–41. 10.1210/er.2012-1003 22829562 PMC3461136

[14] Ganie MA Marwaha RK Aggarwal R Singh S. High prevalence of polycystic ovary syndrome characteristics in girls with euthyroid chronic lymphocytic thyroiditis: a case-control study. Eur J Endocrinol. (2010) 162:1117–22. 10.1530/EJE-09-1012 20332127

[15] Gaberšček S Zaletel K Schwetz V Pieber T Obermayer-Pietsch B Lerchbaum E. Mechanisms in endocrinology: thyroid and polycystic ovary syndrome. Eur J Endocrinol. (2015) 172:R9–21. 10.1530/EJE-14-0295 25422352

[16] Ulrich J Goerges J Keck C Müller-Wieland D Diederich S Janssen OE. Impact of autoimmune thyroiditis on reproductive and metabolic parameters in patients with polycystic ovary syndrome. Exp Clin Endocrinol Diabetes. (2018) 126:198–204. 10.1055/s-0043-110480 29506313

[17] Rosenfield RL. Clinical review: identifying children at risk for polycystic ovary syndrome. J Clin Endocrinol Metab. (2007) 92:787–96. 10.1210/jc.2006-2012 17179197

[18] Idkowiak J Lavery GG Dhir V Barrett TG Stewart PM Krone N Premature adrenarche: novel lessons from early onset androgen excess. Eur J Endocrinol. (2011) 165:189–207. 10.1530/EJE-11-0223 21622478

[19] Voutilainen R Jääskeläinen J. Premature adrenarche: etiology, clinical findings, and consequences. J Steroid Biochem Mol Biol. (2015) 145:226–36. 10.1016/j.jsbmb.2014.06.004 24923732

[20] Kanaka-Gantenbein C Mastorakos G Chrousos GP. Endocrine-related causes and consequences of intrauterine growth retardation. Ann N Y Acad Sci. (2003) 997:150–7. 10.1196/annals.1290.017 14644821

[21] Utriainen P Jääskeläinen J Romppanen J Voutilainen R. Childhood metabolic syndrome and its components in premature adrenarche. J Clin Endocrinol Metab. (2007) 92:4282–5. 10.1210/jc.2006-2412 17698912

[22] Livadas S Dracopoulou M Vasileiadi K Lazaropoulou C Magiakou MA Xekouki P Elevated coagulation and inflammatory markers in adolescents with a history of premature adrenarche. Metab Clin Exp. (2009) 58:576–81. 10.1016/j.metabol.2008.12.002 19303981

[23] Utriainen P Laakso S Liimatta J Jääskeläinen J Voutilainen R. Premature adrenarche–a common condition with variable presentation. Horm Res Paediatr. (2015) 83:221–31. 10.1159/000369458 25676474

[24] Neville KA Walker JL. Precocious pubarche is associated with SGA, prematurity, weight gain, and obesity. Arch Dis Child. (2005) 90:258–61. 10.1136/adc.2004.053959 15723910 PMC1720316

[25] Dayan CM Daniels GH. Chronic autoimmune thyroiditis. N Engl J Med. (1996) 335:99–107. 10.1056/NEJM199607113350206 8649497

[26] Rotondi M de Martinis L Coperchini F Pignatti P Pirali B Ghilotti S Serum negative autoimmune thyroiditis displays a milder clinical picture compared with classic Hashimoto's Thyroiditis. Eur J Endocrinol. (2014) 171:31–6. 10.1530/EJE-14-0147 24743395

[27] Marshall WA Tanner JM. Variations in pattern of pubertal changes in girls. Arch Dis Child. (1969) 44:291–303. 10.1136/adc.44.235.291 5785179 PMC2020314

[28] Bacopoulou F Efthymiou V Landis G Rentoumis A Chrousos GP. Waist circumference, waist-to-hip ratio and waist-to-height ratio reference percentiles for abdominal obesity among Greek adolescents. BMC Pediatr. (2015) 15:50. 10.1186/s12887-015-0366-z 25935716 PMC4446827

[29] Cutfield WS Jefferies CA Jackson WE Robinson EM Hofman PL. Evaluation of HOMA and QUICKI as measures of insulin sensitivity in prepubertal children. Pediatr Diabetes. (2003) 4:119–25. 10.1034/j.1399-5448.2003.t01-1-00022.x 14655269

[30] Greulich WW Pyle SI. Radiographic atlas of skeletal development of the hand and wrist. 2nd ed Stanford, CA: Stanford University Press (1959).

[31] Janssen OE Mehlmauer N Hahn S Offner AH Gärtner R. High prevalence of autoimmune thyroiditis in patients with polycystic ovary syndrome. Eur J Endocrinol. (2004) 150:363–9. 10.1530/eje.0.1500363 15012623

[32] Garelli S Masiero S Plebani M Chen S Furmaniak J Armanini D High prevalence of chronic thyroiditis in patients with polycystic ovary syndrome. Eur J Obstet Gynecol Reprod Biol. (2013) 169:248–51. 10.1016/j.ejogrb.2013.03.003 23548659

[33] Arora S Sinha K Kolte S Mandal A. Endocrinal and autoimmune linkage: evidences from a controlled study of subjects with polycystic ovarian syndrome. J Hum Reprod Sci. (2016) 9:18–22. 10.4103/0974-1208.178636 27110073 PMC4817282

[34] Al-Saab R Haddad S. Detection of thyroid autoimmunity markers in euthyroid women with polycystic ovary syndrome: a case-control study from Syria. Int J Endocrinol Metab. (2014) 12:e17954. 10.5812/ijem.17954 25237328 PMC4166006

[35] Karaköse M Hepsen S Çakal E Saykı Arslan M Tutal E Akın Ş Frequency of nodular goiter and autoimmune thyroid disease and association of these disorders with insulin resistance in polycystic ovary syndrome. J Turk Ger Gynecol Assoc. (2017) 18:85–9. 10.4274/jtgga.2016.0217 28400351 PMC5458441

[36] Singla R Gupta Y Khemani M Aggarwal S. Thyroid disorders and polycystic ovary syndrome: an emerging relationship. Indian J Endocrinol Metab. (2015) 19:25–9. 10.4103/2230-8210.146860 25593822 PMC4287775

[37] Ibáñez L Potau N Zampolli M Riqué S Saenger P Carrascosa A. Hyperinsulinemia and decreased insulin-like growth factor-binding protein-1 are common features in prepubertal and pubertal girls with a history of premature pubarche. J Clin Endocrinol Metab. (1997) 82:2283–8. 10.1210/jcem.82.7.4084 9215308

[38] Ibañez L Potau N Virdis R Zampolli M Terzi C Gussinyé M Postpubertal outcome in girls diagnosed of premature pubarche during childhood: increased frequency of functional ovarian hyperandrogenism. J Clin Endocrinol Metab. (1993) 76:1599–603. 10.1210/jcem.76.6.8501168 8501168

[39] Güven A Cinaz P Bideci A. Is premature adrenarche a risk factor for atherogenesis? Pediatr Int. (2005) 47:20–5. 10.1111/j.1442-200x.2004.02006.x 15693861

[40] Kaloumenou I Mastorakos G Alevizaki M Duntas LH Mantzou E Ladopoulos C Thyroid autoimmunity in schoolchildren in an area with long-standing iodine sufficiency: correlation with gender, pubertal stage, and maternal thyroid autoimmunity. Thyroid. (2008) 18:747–54. 10.1089/thy.2007.0370 18631003

[41] Kabelitz M Liesenkötter KP Stach B Willgerodt H Stäblein W Singendonk W The prevalence of anti-thyroid peroxidase antibodies and autoimmune thyroiditis in children and adolescents in an iodine replete area. Eur J Endocrinol. (2003) 148:301–7. 10.1530/eje.0.1480301 12611610

[42] Stichel H l'Allemand D Grüters A. Thyroid function and obesity in children and adolescents. Horm Res. (2000) 54:14–9. 10.1159/000063431 11182630

[43] Marzullo P Minocci A Tagliaferri MA Guzzaloni G Di Blasio A De Medici C Investigations of thyroid hormones and antibodies in obesity: leptin levels are associated with thyroid autoimmunity independent of bioanthropometric, hormonal, and weight-related determinants. J Clin Endocrinol Metab. (2010) 95:3965–72. 10.1210/jc.2009-2798 20534769

[44] Radetti G Kleon W Buzi F Crivellaro C Pappalardo L di Iorgi N Thyroid function and structure are affected in childhood obesity. J Clin Endocrinol Metab. (2008) 93:4749–54. 10.1210/jc.2008-0823 18840640

[45] Ruszała A Wójcik M Starzyk JB. The impact of thyroid function on the occurrence of metabolic syndrome in obese children and adolescents. Pediatr Endocrinol Diabetes Metab. (2019) 25:1–5. 10.5114/pedm.2019.84705 31343126

[46] Ong KK Kuh D Pierce M Franklyn JA. Medical research council national survey of health and development scientific and data collection teams. Childhood weight gain and thyroid autoimmunity at age 60-64 years: the 1946 British birth cohort study. J Clin Endocrinol Metab. (2013) 98:1435–42. 10.1210/jc.2012-3761 23436917 PMC3651609

[47] Hersoug LG Linneberg A. The link between the epidemics of obesity and allergic diseases: does obesity induce decreased immune tolerance? Allergy. (2007) 62:1205–13. 10.1111/j.1398-9995.2007.01506.x 17845592

[48] Asvold BO Bjøro T Vatten LJ. Association of serum TSH with high body mass differs between smokers and never-smokers. J Clin Endocrinol Metab. (2009) 94:5023–7. 10.1210/jc.2009-1180 19846737

[49] Bhowmick SK Dasari G Levens KL Rettig KR. The prevalence of elevated serum thyroid-stimulating hormone in childhood/adolescent obesity and of autoimmune thyroid diseases in a subgroup. J Natl Med Assoc. (2007) 99:773–6. PMID: 17668643 PMC2574343

[50] Jin HY. Prevalence of subclinical hypothyroidism in obese children or adolescents and association between thyroid hormone and the components of metabolic syndrome. J Paediatr Child Health. (2018) 54:975–80. 10.1111/jpc.13926 29768692

[51] Knudsen N Laurberg P Rasmussen LB Bülow I Perrild H Ovesen L Small differences in thyroid function may be important for body mass index and the occurrence of obesity in the population. J Clin Endocrinol Metab. (2005) 90:4019–24. 10.1210/jc.2004-2225 15870128

[52] Loviselli A Velluzzi F Mossa P Cambosu MA Secci G Atzeni F; sardinian schoolchildren study group. The sardinian autoimmunity study: 3. Studies on circulating antithyroid antibodies in sardinian schoolchildren: relationship to goiter prevalence and thyroid function. Thyroid. (2001) 11:849–57. 10.1089/105072501316973109 11575854

[53] Witkowska-Sędek E Kucharska A Rumińska M Pyrżak B. Thyroid dysfunction in obese and overweight children. Endokrynol Pol. (2017) 68:54–60. 10.5603/EP.2017.0007 28255980

[54] Drobniak A Kanecki K Grymowicz M Radowicki S. Serum leptin concentration in women of reproductive age with euthyroid autoimmune thyroiditis. Gynecol Endocrinol. (2016) 32:128–31. 10.3109/09513590.2015.1092512 26440361

[55] Kennedy A Gettys TW Watson P Wallace P Ganaway E Pan Q The metabolic significance of leptin in humans: gender-based differences in relationship to adiposity, insulin sensitivity, and energy expenditure. J Clin Endocrinol Metab. (1997) 82:1293–300. 10.1210/jcem.82.4.3859 9100610

[56] MacIver NJ Thomas SM Green CL Worley G. Increased leptin levels correlate with thyroid autoantibodies in nonobese males. Clin Endocrinol (Oxf). (2016) 85:116–21. 10.1111/cen.12963 26445359

[57] Duntas LH Biondi B. The interconnections between obesity, thyroid function, and autoimmunity: the multifold role of leptin. Thyroid. (2013) 23:646–53. 10.1089/thy.2011.0499 22934923

[58] Kiernan K MacIver NJ. The role of the adipokine leptin in immune cell function in health and disease. Front Immunol. (2021) 11:622468. 10.3389/fimmu.2020.622468 33584724 PMC7878386

[59] Wang S Baidoo SE Liu Y Zhu C Tian J Ma J T cell-derived leptin contributes to increased frequency of T helper type 17 cells in female patients with Hashimoto's Thyroiditis. Clin Exp Immunol. (2013) 171:63–8. 10.1111/j.1365-2249.2012.04670.x 23199324 PMC3530096

[60] Mazaheri T Sharifi F Kamali K. Insulin resistance in hypothyroid patients under levothyroxine therapy: a comparison between those with and without thyroid autoimmunity. J Diabetes Metab Disord. (2014) 13:103. 10.1186/s40200-014-0103-4 25364704 PMC4216656

[61] Cunha CA Neves C Neves JS Oliveira SC Sokhatska O Dias C Cardiovascular risk factors in patients with autoimmune thyroiditis. Rev Port Endocrinol Diabetes Metab. (2017) 12:133–41. 10.1530/endoabs.49.GP199

[62] Zhao H Zhang Y Ye J Wei H Huang Z Ning X A comparative study on insulin secretion, insulin resistance and thyroid function in patients with polycystic ovary syndrome with and without Hashimoto's Thyroiditis. Diabetes Metab Syndr Obes. (2021) 14:1817–21. 10.2147/DMSO.S300015 33953581 PMC8089092

[63] Ibáñez L Potau N Chacon P Pascual C Carrascosa A. Hyperinsulinaemia, dyslipaemia and cardiovascular risk in girls with a history of premature pubarche. Diabetologia. (1998) 41:1057–63. 10.1007/s001250051030 9754824

[64] Sopher AB Jean AM Zwany SK Winston DM Pomeranz CB Bell JJ Bone age advancement in prepubertal children with obesity and premature adrenarche: possible potentiating factors. Obesity (Silver Spring). (2011) 19:1259–64. 10.1038/oby.2010.305 21311512 PMC3637026

[65] Kassi E Pervanidou P Kaltsas G Chrousos G. Metabolic syndrome: definitions and controversies. BMC Med. (2011) 9:48. 10.1186/1741-7015-9-48 21542944 PMC3115896

[66] Cizza G Dorn LD Lotsikas A Sereika S Rotenstein D Chrousos GP. Circulating plasma leptin and IGF-1 levels in girls with premature adrenarche: potential implications of a preliminary study. Horm Metab Res. (2001) 33:138–43. 10.1055/s-2001-14927 11355746

[67] Dittrich R Kajaia N Cupisti S Hoffmann I Beckmann MW Mueller A. Association of thyroid-stimulating hormone with insulin resistance and androgen parameters in women with PCOS. Reprod Biomed Online. (2009) 19:319–25. 10.1016/s1472-6483(10)60165-4 19778476

[68] Mueller A Schöfl C Dittrich R Cupisti S Oppelt PG Schild RL Thyroid-stimulating hormone is associated with insulin resistance independently of body mass index and age in women with polycystic ovary syndrome. Hum Reprod. (2009) 24:2924–30. 10.1093/humrep/dep285 19654109

